# Multi-drug resistance of *Staphylococcus aureus *Strains in Baqiyatallah hospital: a Primary Step Towards Digital Health Biomonitoring Systems

**DOI:** 10.22037/ijpr.2020.112966.14042

**Published:** 2020

**Authors:** Ahmadreza Katoziyan, Abbas Ali Imani Fooladi, Ramezan Ali Taheri, Saba Vatanpour

**Affiliations:** a *Center of Excellence in Phylogeny of Living Organisms, School of Biology, University of Tehran, Iran. *; b *Applied Microbiology Research Center, Systems Biology and Poisonings Institute, Baqiyatallah University of Medical Sciences, Tehran, Iran.*; c *Nanobiotechnology Research Center, Baqiyatallah University of Medical Sciences, Tehran, Iran. *; d *Department of Biology, University of British Columbia, Vancouver, Canada.*

**Keywords:** Multiple drug resistance, Staphylococcus aureus, Nosocomial infections, Cryptic specie, Genetic resistance

## Abstract

The aim of the study was to evaluate the drug-resistance patterns of *Staphylococcus aureus* infections in Baqiyatallah hospital within 2010–2019 and to present a novel monitoring and detection system making use of molecular laboratory methods teamed with molecular delimitation analyses. This in turn is a primary step to establishment of a digital health system within Baqiyatallah hospital as a perfect pilot instance for other hospitals to follow upon. Totally, 100 patients of Baqiyatallah hospital suspicious of *Staphylococcus aureus* infections were sampled. Bacterial identity confirmations were done using routine biochemical test. Antibiograms were made for all the patients in this study. Consequently, bacterial total DNA was extracted and 16S rDNA gene amplified and sequenced for all patients. To uncover any cryptic strain grouping within the samples, a molecular delimitation method, i.e. automated barcode gap discovery (ABGD), was done. Our results showed Ceftaroline to be the most and Erythromycin and Oxacillin the least effective drugs. Delimitation uncovered 19 groups out of which group 19 seemed to have location-specific genetic signals in regards to susceptibility of Erythromycin and Oxacillin. Our results indicate the importance of genetic identification of bacteria with respect to their genetic patterns before antibiotic administration in order to both reduce unnecessary medicine use and to biomonitor the bacterial patterns in respect to their behavior towards general antibiotics.

## Introduction

Multidrug resistance has become a serious global issue within recent decades (1, 2). Many bacterial infections have become irresponsive to commonly used antibiotics, making the treatment process as well as choice of antibiotics a challenging one to both patients and physicians. This is even more critical in case of hospital-acquired infections (3).

Infections acquired in hospitals became a major health concern during the culmination of antibiotics application. Owing to the mentioned infections both the usage of antibiotics and the costs for extended hospitalization increase dramatically (4, 5). The excessive use of antibiotics could be systemic, i.e. within hospitals, or individual-based, i.e. patients using the antibiotics on their own account. Thus, it has been advised to that general physicians should consult infectious disease specialists before they try to prescribe any type of antibiotics (6). Also, more general information must be provided to the public to inform them about the consequences of using unprescribed medicine. Furthermore, exploring new sources of potentially medically useful substances including scorpion venoms have also been suggested (7).

*S. aureus* is known as one of the most important types of pathogens involved in the hospital-acquired infections (8-10). This bacterium is Gram-positive and round-shaped, it does not form spores and is facultatively anaerobic (11). Normally, hospitalized patients with lowered immunity systems are more prone to these types of infections. *S. aureus* can affect superficial and deep tissues in addition to the local abscess lesion (12). Virulence modes of *S. aureus *comprise toxins, enzymes, as well as immune system modulators (13).  

Recently, Many bacterial species are found to be cryptic (14). To uncover these, especially the pathogenic ones which play important roles in hospital infections it is necessary to first barcode the samples (15). To do so, the 16S rRNA gene of these organisms is amplified using universal primers (16). This gene has proved to be quite useful in identifying bacterial strains(17). The barcodes are then tested against a databank and hence identified. These results combined with robust molecular species delimitation methods such as Statistical parsimony (SP) (18), automated barcode gap discovery (ABGD) (19), bayesian generalized mixed Yule coalescent (bGMYC) (20),  and Bayesian Poisson tree processes (bPTP) (21) would clarify and uncover the identity of the cryptic species causing hospital-acquired infections (22). Employing these methods eventually leads to a better understanding of infectious bacteria behavior especially, to antibiotics and would be a first step in employing efficient biosurveillance within hospital wards. 

## Experimental

A total of 100 patients from Baqiyatallah hospital suspicious of *Staphylococcus aureus* infections were sampled within this study. The samples were taken from different sources including wound discharge, urine, pleural fluid, blood, and BAL (Bronchoalveolar lavage). 

Taken samples were transferred and cultured in Baqiyatallah Central microbiology laboratory. The cultures were made on differential Mannitol Salt Agar (MSA) medium and incubated at 37 °C for 24-48 h. Also, primary identification of the Staphylococcus bacteria was made using basic routine macroscopic and biochemical tests. 

The antibiotic resistance patterns of isolates were evaluated using disc diffusion. The following antibiotics were used: Clindamycin (CC; 2 μg), Ceftaroline (CPT; 30 μg), Cefazoline (CZ; 30 μg), Doxycline (D; 30 μg), Erythromycin (E; 15 μg), Oxacillin (OX; 1 μg), Rifampicin (RA; 5 μg), Cotrimoxazole (SXT; 1.25/23.75 μg), Tetracycline (TE; 30 μg), and Teicoplanin (TEC; 30 μg). 

DNA extraction was done using Qiagen DNeasy 96 Blood & Tissue Kit from the collected colonies. The full length of 16S ribosomal RNA gene was amplified using the primer pair 8F (AGAGTTTGATCCTGGCTCAG) and 1492R (CGGTTACCTTGTTACGACTT). Polymerase chain reaction (PCR) was carried out in a total volume of 25μL containing 2.5μL 10X PCR buffer, 2.5μL dNTPs (2mM), 0.125μL of each primer (100 pmol/μL), 0.125μL of Hotmaster Taq (5 U/μL, 5 PRIME GmbH, Hamburg, Germany), 1μL of template DNA (20–60 ng/μL) and molecular grade water. The PCR setting was: initial denaturation at 94 °C for 60 s; 36 cycles of denaturation at 94 °C for 30 s, annealing at 49 °C for 45 s, extension at 65 °C for 60 s; final extension at 65 °C for 5 min. For DNA sequencing, 10μL of PCR product was enzymatically purified with 0.5μL ExoI (20U/μL) and 1μL FastAP (1U/μL) (both Thermo Fisher Scientific, Schwerte, Germany).

The reaction mix was incubated at 37 °C for 25 min and 85 °C for 15 min. The purified products were sequenced at Macrogen Inc. (Korea) or on an ABI 3130xl sequencer (University of Tehran). Sequence chromatograms were edited and assembled in Geneious 8.1.9 (23). The sequences were BLASTED to make sure that they were contamination-free and that they belonged to *S. aureus* species. The 16S rRNA-alignment was constructed using the MUSCLE algorithm plugin in Geneious with eight iterations (24). The Neighbor-joining trees were made using MEGA X (25).

We used the Automatic Barcode Gap Discovery (ABGD) delimitation approach to uncover any potential cryptic species within our samples. This method is based upon pairwise genetic distance calculations. The sequences are semi-automatically grouped in a way that distances between sequences of two groups are always larger than the barcode gap value (19). We tested our dataset with a combination of ABGD settings within the parameter range of Pmin = 0.001, Pmax = 0.08–0.10, and gap width = 0.1–0.5. A Kimura-2-parameter (K2P) corrected genetic distance matrix was used as it is the standard model proposed for DNA barcoding analyses. K2P-distances were calculated using MEGA X (25).

## Result and Discussion

Final dataset in this study included 61 men and 39 women. All cultures and biochemical tests on the 100 gathered samples showed the infections to be due to *Staphylococcus aureus*. Table 1 shows the sources of infections sampled in this study.

The results of antibiogram screening of the colonies of *Staphylococcus aureus* are shown in Table 2. According to these results, Ceftaroline was the only completely effective antibiotic on *Staphylococcus aureus* infections. On the other hand, the recovered *Staphylococcus aureus* strains were least sensitive to Erythromycin (58%) and Oxacillin (69%). Given the fact that both Oxacillin and Erythromycin have been in use extensively and for a long time within hospitals all over the world as well as Baqiyatallah hospital (10, 26, 27), it was expected to observe the most resistance in them. This has been the case with many other long-used antibiotics both locally (10, 28-30) and globally (31-33). Conversely, Ceftaroline is a 5^th^ generation Cephalosporin with a wide range of action on gram-positive bacteria. This antibiotic has only been introduced in 2011 and has in application within Baqiyatallah hospital for a short time (26) which in turn has refused to provide the opportunity to *Staphylococcus aureus* strains to develop resistance to it (34). 

Interestingly, when considering gender-based response to the applied antibiotics, females and males were most responsive to Ceftaroline and least responsive to Co-Trimoxazole and Tetracycline, respectively (Table 3). This could be considered as a differential response of the patients to different antibiotics (35). However, this result must be treated with caution and used as foundation for further studies into such differential responses (35, 36). 

**Figure 1:**
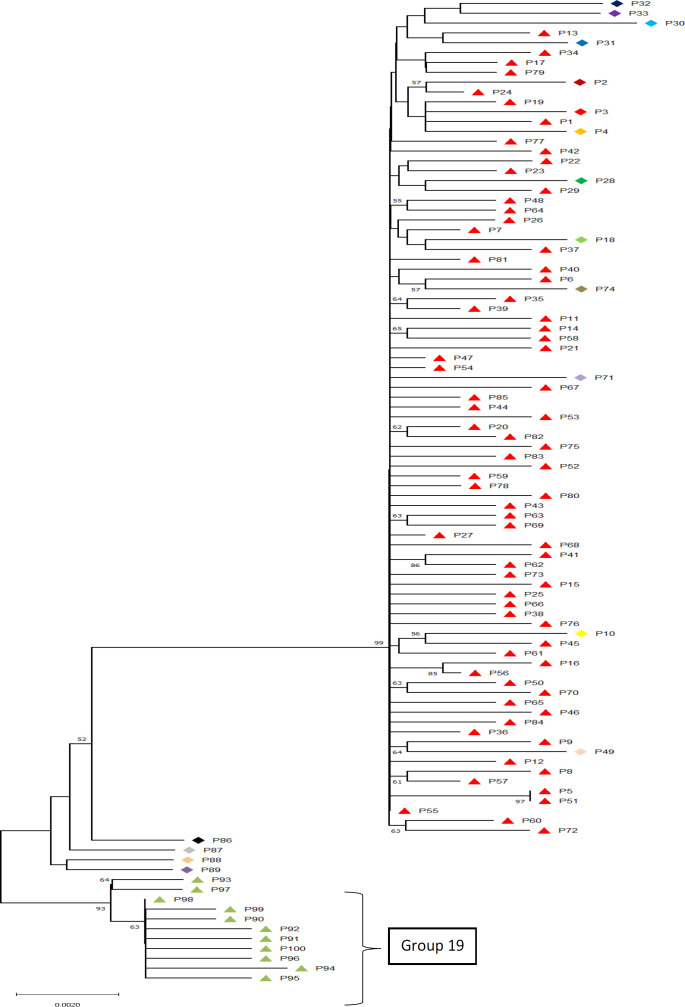
The evolutionary history was inferred using the Neighbor-Joining method (39). The percentage of replicate trees in which the associated taxa clustered together in the bootstrap test (2000 replicates) are shown next to the branches (40). The results of ABGD delimitation method is shown next to samples

Bacteria have a great potential to evolve through random mutations, developing resistance to the risk and fatal factors in their environment, including drug-resistance (1). Also, it has been shown that specific alterations in specific places on genes including 16S rDNA which plays a role in the formation of the ribosome complex in bacteria could potentially lead to such immunities (14, 16, 37). Bacterial identification via molecular delimitation methods using DNA barcoding in order to recover cryptic species or specific genetic patterns has proven to be of extreme worth so as to explain differential behaviors of organisms (38). Our molecular delimitation results showed that among the sampled patients at least 19 distinct genetically unique groups were identifiable (Figure 1). These unique genetic identities show a maximum difference of 1.7% and an overall average difference of 1%. Also, the delimitation results showed that the barcode gap falls within the 1% difference among the samples. This proves that although all the studied organisms are to be considered as *Staphylococcus aureus*, they can be categorized into different groups. 

Although, group 1 to 18 did not show any specific patterns of resistance in relation to their genetic variations in 16s rRNA region, group 19 showed specific nucleotide alterations at constant locations. These main differences with other sequences were at positions 447, 746-7, 1006-7, 1090-1, 1152-4, and 1247-8. These constant changes combined with the fact that unlike other genetic identities, group 19 specifically shows dominant sensitivity to the commonly resisted antibiotics including Oxacillin and Erythromycin leads to consideration of the possibility of the role of these specific variations in the development of resistant *Staphylococcus aureus* strains. Such specifically nucleotide-related characteristics have been found in other bacteria (41-44); however, we are reporting these locations for the first time in Iranian hospitals as possible antibiotic resistance-causing nucleotide regions in 16s rRNA. This pattern seems very useful for biomonitoring and diagnosis plans (45) within hospitals generally and within Baqiyatallah hospital in specific as group 19 patients showed the highest sensitivity to both Erythromycin and Oxacillin which otherwise were the least effective antibiotics in this study. 

**Table 1:** Origin of the infections studied

		**Frequency**	**Percent**	**Cumulative Percent**
Origin of infection	BAL	17	17.0	17.0
	Blood	53	53.0	70.0
	Pleural Fluid	1	1.0	71.0
	Urine	13	13.0	84.0
	Wound Discharge	16	16.0	100.0
	Total	100	100.0	

**Table 2:** Antibiogram results in all patients. CPT is the most effective while E and OX are the most resisted

**Antibiotic**	**Sensitive**		**Resistant**		**Total**
	Table N %	Standard Error of Table N %	Table N %	Standard Error of Table N %	Table N %
Clindamycin (CC)	75.0%	4.3%	25.0%	4.3%	100.0%
Ceftaroline (CPT)	100.0%	.	0.0%	.	100.0%
Cefazolin (CZ)	70.0%	4.6%	30.0%	4.6%	100.0%
Doxycycline (D)	85.0%	3.6%	15.0%	3.6%	100.0%
Erythromycin (E)	58.0%	4.9%	42.0%	4.9%	100.0%
Oxacillin (OX)	69.0%	4.6%	31.0%	4.6%	100.0%
Rifampicin (RA)	73.0%	4.4%	27.0%	4.4%	100.0%
Co-Trimoxazole (SXT)	87.0%	3.4%	13.0%	3.4%	100.0%
Tetracycline (TE)	89.0%	3.1%	11.0%	3.1%	100.0%
Teicoplanin (TEC)	71.0%	4.5%	29.0%	4.5%	100.0%

**Table 3:** Antibiotic sensitivity of cultures based on patient’s gender. N% represents the percentage of the number of patients within the categories. Where the Standard Error was not computable the cells were left blank

**Antibiotic**		**Gender**			
		**Male**		**Female**		
		N %	Standard Error of N %	N %	Standard Error of N %	
Clindamycin (CC)	Sensitive	57.3%	5.7%	42.7%	5.7%	
	Intermediate	0.0%	.	0.0%	.	
	Resistant	72.0%	9.0%	28.0%	9.0%	
Ceftaroline (CPT)	Sensitive	61.0%	4.9%	39.0%	4.9%	
	Intermediate	0.0%	.	0.0%	.	
	Resistant	0.0%	.	0.0%	.	
Cefazolin (CZ)	Sensitive	55.7%	5.9%	44.3%	5.9%	
	Intermediate	0.0%	.	0.0%	.	
	Resistant	73.3%	8.1%	26.7%	8.1%	
Doxycycline (D)	Sensitive	57.6%	5.4%	42.4%	5.4%	
	Intermediate	0.0%	.	0.0%	.	
	Resistant	80.0%	10.3%	20.0%	10.3%	
Erythromycin (E)	Sensitive	60.3%	6.4%	39.7%	6.4%	
	Intermediate	0.0%	.	0.0%	.	
	Resistant	61.9%	7.5%	38.1%	7.5%	
Oxacillin (OX)	Sensitive	59.4%	5.9%	40.6%	5.9%	
	Intermediate	0.0%	.	0.0%	.	
	Resistant	64.5%	8.6%	35.5%	8.6%	
Rifampicin (RA)	Sensitive	53.4%	5.8%	46.6%	5.8%	
	Intermediate	0.0%	.	0.0%	.	
	Resistant	81.5%	7.5%	18.5%	7.5%	
Co-Trimoxazole (SXT)	Sensitive	62.1%	5.2%	37.9%	5.2%	
	Intermediate	0.0%	.	0.0%	.	
	Resistant	53.8%	13.8%	46.2%	13.8%	
Tetracycline (TE)	Sensitive	57.3%	5.2%	42.7%	5.2%	
	Intermediate	0.0%	.	0.0%	.	
	Resistant	90.9%	8.7%	9.1%	8.7%	
Teicoplanin (TEC)	Sensitive	59.2%	5.8%	40.8%	5.8%	
	Intermediate	0.0%	.	0.0%	.	
	Resistant	65.5%	8.8%	34.5%	8.8%	

## Conclusion

Our study also shows that excessive use of antibiotics would eventually lead to the bacteria gaining resistance mechanisms towards them. However, not all bacteria identified as one specific strain would behave similarly to the same antibiotics; this is mainly due to cryptic groups and strains within these bacteria (e.g. group 19 in our study).

In this study, we iterate the importance of genome-related antibiotic resistance mutations. Such mutations, as shown, provide scientists, hospital managers, and all health-system-decisionmakers with specific items to follow within a hospital-based biosurveillance system to prevent, detect, and treat bacterial infections. Such measures would lead to the establishment of a digital health system which eventually benefits the patients and lessens the burden on the health system by decreasing the time patients staying in hospitals through reliably predicting bacterial behavior to commonly used antibiotics. 
